# The infrapatellar fat pad–synovium immunometabolic interface in knee osteoarthritis: a local amplifier of joint inflammation

**DOI:** 10.3389/fimmu.2026.1977398

**Published:** 2026-09-21

**Authors:** Huacheng Wei, Chendeng Lao, Ronghe Gu

**Affiliations:** 1 Jinan University, Guangzhou, China; 2 Department of Orthopedics and Joint Surgery, The First People’s Hospital of Nanning, Nanning, China

**Keywords:** adipokine, fibroblast, immunometabolism, infrapatellar fat pad, knee osteoarthritis, macrophage, synovium

## Abstract

Knee osteoarthritis (OA) is a whole-joint disease in which synovitis is associated with pain and structural progression. The infrapatellar fat pad (IPFP) lies next to the anterior synovium and contains adipocytes, mesenchymal and fibroblast-like stromal cells, macrophages, vascular cells, and nerves. Alongside its mechanical cushioning role, the IPFP releases adipokines, cytokines, chemokines, lipid mediators, and extracellular vesicles. Adipocyte stress, matrix remodeling, fibrosis, and immune cell accumulation alter this secretory profile in OA. Single-cell analyses of paired human tissues suggest that the IPFP and synovium may share mesenchymal progenitors and undergo coordinated inflammatory and profibrotic remodeling. We propose that these tissues form an immunometabolic interface in which macrophages integrate lipid and damage signals, while stromal cells recruit immune cells and remodel the extracellular matrix (ECM). These interactions may link local metabolic dysfunction to persistent synovitis, cartilage catabolism, and pain. Human evidence, however, comes largely from cross-sectional studies of tissues collected during surgery for advanced OA. Computational predictions of cell-cell communication require functional testing, and animal interventions cannot establish causality in humans. The interface is therefore best regarded as a potential local amplifier of inflammation; its role in disease initiation remains unproven. Longitudinal studies with spatial resolution and interventions stratified by phenotype are needed to determine when, in which patients, and through which components the interface has therapeutic value.

## 1 Introduction

Osteoarthritis (OA) involves more than passive cartilage wear. Cartilage, synovium, subchondral bone, adipose tissue, menisci, immune cells, and sensory nerves all contribute to the dynamic intra-articular environment (1). Synovitis is associated with pain and, in some cohorts, structural deterioration. Its extent and biology vary among patients and disease stages (2, 3), so inflammation should not be viewed as a uniform feature of knee OA.

The infrapatellar fat pad (IPFP) is located within the fibrous joint capsule but outside the synovial cavity; its joint-facing surface is covered by the synovial lining. The IPFP is continuous with the anterior synovium and has a rich supply of blood vessels and nerves, as well as a diverse cellular composition. These features make a strict biological boundary between the two tissues difficult to define (4, 5). The IPFP absorbs and redistributes mechanical loads while also releasing adipokines, cytokines, chemokines, lipids, and extracellular vesicles. OA-associated fibrosis, adipocyte dysfunction, and immune cell remodeling may therefore alter signals transmitted to the adjacent synovium (4, 6).

Previous studies have often considered IPFP inflammation, adipokines, synovitis, macrophages, and cartilage degeneration separately. Recent single-cell studies in humans and mice support an integrated tissue unit model (5–7). Mesenchymal cell populations span adipogenic and synovial fibroblast differentiation trajectories, while macrophage and stromal cell programs undergo coordinated changes in OA (7). Here, an immunometabolic interface means reciprocal coupling between lipid storage, release, and processing, cellular metabolism, and immune–stromal activity across the IPFP and synovium. It requires evidence that altered metabolism affects cells in the adjacent tissue; shared cytokine expression or immune infiltration alone is insufficient. This distinction can be tested by tracing tissue-derived metabolites or selectively perturbing metabolism and measuring recipient-cell responses.

## 2 The IPFP as an immunometabolic niche

The IPFP consists of mature adipocytes and a vascularized stromal region (4, 8). The stromal region contains mesenchymal progenitor cells, fibroblast-like cells, endothelial cells and mural cells, macrophages, lymphocytes, and neural components (9, 10). This composition enables the IPFP to simultaneously sense mechanical strain, metabolic stress, tissue damage, and immune signals. Histological studies of OA IPFP report changes in adipocyte size, immune cell infiltration, vascular features, and fibrosis. The extent of these changes varies with sampling location, disease stage, obesity status, and the choice of control tissue (8, 9).

In a paired atlas of the IPFP and synovium, Tang et al. identified DPP4-positive mesenchymal cells as a predicted common progenitor source for IPFP adipocytes and synovial lining fibroblasts. The atlas linked OA to inflammatory and profibrotic mesenchymal cell states, including BGN-positive intermediate fibroblasts, and to APOE-associated fibroblast-macrophage communication (5). Peters et al. sequenced 82,924 nuclei from 21 IPFP samples using single-nucleus RNA sequencing, complemented by spatial transcriptomics and metabolomics of cultured fibroblast supernatants. They identified diverse cell states and fibroblast transcriptomic differences associated with OA, sex, and obesity (6). Together, these datasets resolve OA-associated changes by cell state and location.

Adipocyte metabolic state alters the balance among leptin, adiponectin, free fatty acids, oxylipins, and redox signaling. Stromal cells produce IL-6, chemokines, matrix proteins, and profibrotic factors; macrophages respond to lipids and damaged matrix, and can reciprocally remodel stromal cell behavior. IPFP secretion therefore reflects interactions among several cell types. Its biological significance for OA depends on whether these products can reach the synovium at effective concentrations, act locally via paracrine or matrix-bound mechanisms, or enter the synovial fluid to influence other joint tissues.

Vascular, neural, and extracellular matrix (ECM) compartments further contribute to regulation. Blood vessels allow leukocytes to enter the tissue and expose local cells to the systemic metabolic environment. A higher body mass index, however, does not necessarily correspond to a consistent inflammatory profile within the IPFP. Fibrous septa can facilitate or restrict molecular diffusion, while matrix fragments can signal through innate immune receptors. Given the rich sensory innervation, neuropeptides released upon nociceptor activation may alter vascular permeability and immune cell behavior. Epigenomic profiling of the IPFP and circular RNA analysis of the IPFP–synovium unit identify additional regulatory changes associated with OA (11, 12). Anatomical evidence is consistent with this model, but the contributions of individual compartments at different disease stages have not been quantified (13).

## 3 Cellular crosstalk at the IPFP–synovium interface

Macrophages can integrate metabolic and inflammatory signals. Both OA synovium and IPFP contain abundant macrophages, whose mixed marker expression, tissue-resident characteristics, and functional plasticity are poorly captured by the M1/M2 dichotomy. In end-stage human OA tissue, Hengtrakool et al. found a predominant CD11c+/CD206+ macrophage phenotype in both tissues. Conditioned media from either IPFP or synovium also induced distinct macrophage phenotypes *in vitro* (14). These findings support local conditioning of macrophage states, but do not identify a stable pathogenic subset. Single-cell data have similarly revealed inflammatory, lipid-handling, and resident-like states (5, 6), while tissue-remodeling programs vary with tissue origin and disease context (15).

Macrophage recruitment may be sustained by CCL2 and related chemokines released by stressed stromal cells, adipocytes, or damaged joint tissues. In experimental OA, CCL2/CCR2, but not CCL5/CCR5, mediates monocyte recruitment, inflammation, and cartilage destruction, indicating pathway selectivity beyond a nonspecific increase in chemokines (16). Upon entering the tissue, macrophages can release cytokines, growth factors, proteases, and lipid mediators that influence angiogenesis and nociceptive signaling. Aberrant lipid uptake, mitochondrial stress, or impaired clearance of dead cells may prolong this response. Direct evidence of efferocytosis dysfunction at the human IPFP–synovium interface, however, remains lacking.

Fibroblasts and mesenchymal stromal cells form the structural basis of this network. OA synovium contains functionally distinct fibroblast subpopulations associated with inflammatory signaling and ECM remodeling (17, 18). Additional subsets are linked to fibrosis and chondrocyte injury (19). Lineage inference and mouse tracing studies indicate that IPFP stromal cell populations share some of these programs and can differentiate into adipocytes, lining fibroblasts, and myofibroblasts (5, 7). Their secretion of IL-6, CXCL and CCL chemokines, growth factors, collagen, proteoglycans, and matrix-remodeling enzymes recruits or retains immune cells. Macrophage-derived mediators can, in turn, activate fibroblasts through feedback loops. In a diabetic OA model, targeting YAP1-regulated glycolysis in fibroblast-like synoviocytes reduced macrophage infiltration, linking stromal metabolic programs to myeloid cell recruitment (20). This is evidence for metabolic regulation within the synovium; it does not establish that IPFP-derived signals drive the glycolytic change. Single-cell analysis, fate mapping, and functional experiments implicated macrophage-derived oncostatin M signaling to synovial fibroblasts in OA synovitis (21). Its contribution to IPFP–synovium communication remains to be established.

Adipocyte–immune cell interactions constitute the metabolic component of this loop. Adipocyte hypertrophy is not the only abnormality; impaired lipid storage, altered fatty acid composition, oxidative stress, cellular senescence, and extracellular vesicle cargo can all modify macrophage and fibroblast behavior. In rabbits with experimental OA, a high-fat diet aggravated synovitis alongside loss and remodeling of synovial adipose tissue (22). Extracellular vesicles derived from OA donor IPFP can impair cartilage metabolism and induce chondrocyte senescence in experimental systems (23). In mice, blocking osteopontin–integrin β3 signaling in the IPFP reduced macrophage accumulation, fibrosis, and cartilage damage (24). These interventions support the plausibility of the proposed mechanisms, but do not show that adipocyte dysfunction initiates typical human OA.

These findings suggest a feedback loop. Metabolically stressed adipocytes and remodeling stromal cells alter local soluble and matrix-bound signals, to which macrophages respond by producing inflammatory, fibrotic, and lipid mediators. Activated fibroblasts remodel the ECM and chemokine gradients, altering immune cell residency and adipocyte function. Spatial proximity may allow low-abundance local signals to have biological effects even when their concentrations in peripheral blood are low. The model also includes inflammation resolution: tissue-resident macrophages, stromal cells, and specialized pro-resolving mediators (SPMs) may restore homeostasis following transient injury. OA may involve a failure to terminate this circuit after injury. **Figure 1** summarizes the proposed interactions.

**Figure 1:**
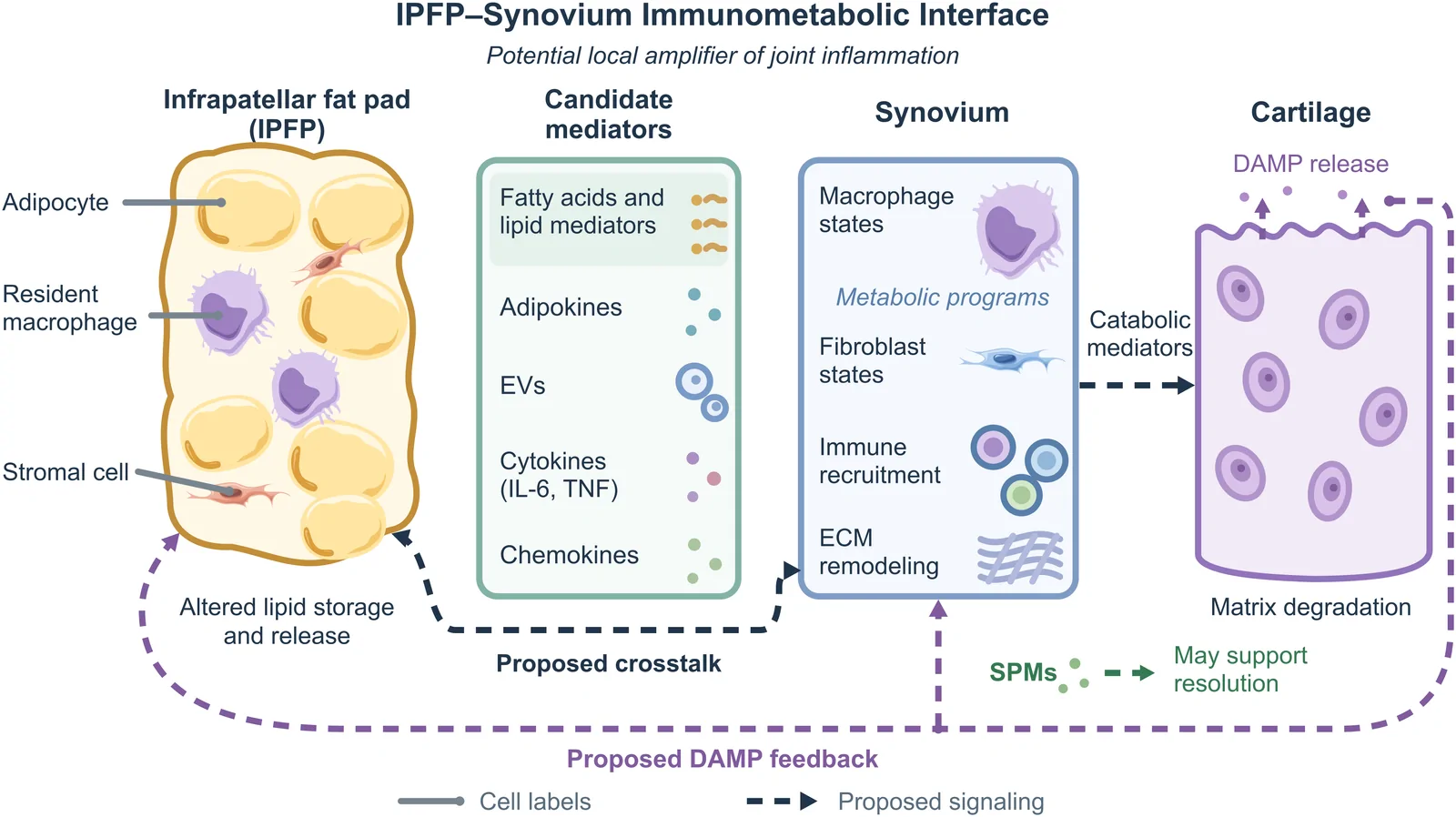
Proposed immunometabolic crosstalk among the infrapatellar fat pad (IPFP), synovium, and cartilage in knee osteoarthritis (OA). Lipid storage and release, lipid mediators, and cellular metabolic programs are linked to immune–stromal activity. Gray dot-ended lines label cells. The navy double-headed dashed path indicates reciprocal IPFP–synovium crosstalk; the navy single-headed path links synovial catabolic mediators to cartilage damage. The purple path represents damage-associated molecular pattern (DAMP) release and feedback; the green path indicates possible resolution by specialized pro-resolving mediators (SPMs). The central mediator panel does not identify tissue sources. All arrows depict proposed pathways, with different levels of evidence, rather than demonstrated transfer or causality in human OA. The interface is a potential local amplifier. Therapeutic evidence largely concerns broader OA/synovitis, and candidate patient strata require validation. ECM, extracellular matrix; EVs, extracellular vesicles; IL-6, interleukin-6; TNF, tumor necrosis factor.

Other immune populations may modulate this circuit. T cells, B cells, mast cells, neutrophils, and innate lymphoid cells occur at low or variable abundances in OA synovium or IPFP. Their intermittent activity after injury may be missed in end-stage samples. Endothelial and mural cells regulate leukocyte entry, while nerves contribute neuro-immune signals.

Experimental approaches capture different parts of the interface. Conditioned media detect secreted factors but remove gradients, cell contact, tension, and matrix sequestration. Tissue dissociation can alter transcriptional states and preferentially lose fragile adipocytes; histology retains location but detects fewer proteins. Organotypic IPFP–synovium co-cultures combined with local sampling or isotope-labeled substrates could test signal origin, transfer, and biologically active concentrations.

## 4 Soluble and metabolic signals linking the IPFP and synovium

Adipokines are common signals between these tissues, but their effects depend on context. Leptin can enhance inflammatory and catabolic responses in synovial fibroblasts and other joint cells. Adiponectin has protective or pro-inflammatory associations that vary with isoform, concentration, cell type, and disease context. Paired human tissue studies show that adipokine expression differs between the synovium and IPFP and varies with metabolic-syndrome status (25). In symptomatic women with knee OA, one study found that synovial adiponectin was more strongly associated with clinical severity than leptin (26). An earlier study found no significant adiponectin association after adjustment, indicating that findings depend on the cohort and analytical model (27). Neither study establishes the tissue of origin or direction of effect. Leptin and adiponectin alone cannot explain the inflammatory and degradative effects of IPFP-conditioned medium on joint cells (28).

Source and context also matter for other adipokines. OA cartilage itself can produce retinol-binding protein 4, which is associated with matrix metalloproteinases. Its presence in synovial fluid therefore does not establish an IPFP origin (29).

Classical cytokines and chemokines link cell recruitment to matrix activation. Several cell types at the interface can produce IL-6, TNF, IL-1β, CCL2, and related mediators, making it difficult to assign them to a single tissue or cell source. Their combined effects may matter more than the concentration of any one molecule: chemokines establish recruitment gradients, cytokines alter macrophage and fibroblast states, and activated cells release further mediators. Human tissue data mainly show correlations. Animal blockade studies targeting CCL2/CCR2 and granulocyte-macrophage colony-stimulating factor (GM-CSF) provide mechanistic evidence in experimental OA (16, 30), while CCL17 blockade provides a further model-dependent example (31). These findings come from broader OA or synovitis models and do not isolate signaling across the IPFP–synovium boundary.

Lipid signaling remains understudied, although it may distinguish this interface from general cytokine networks. Fatty acid profiles in the IPFP and synovial fluid differ between OA and rheumatoid arthritis; targeted lipidomics in human knee OA also shows simultaneous activation of inflammatory and pro-resolving pathways (32, 33). The effects of fatty acids and their derivatives on stromal and immune cells depend on context. Oxylipins and lysophospholipids include inflammatory mediators, whereas SPMs may promote resolution (34). In obese mice, a pro-resolving lipid mediator alleviates the OA phenotype by altering synovial macrophage polarization (35). Diet, obesity, medication, sample processing, and oxidative processes can strongly affect measured lipid abundance. Identifying a lipid as a causal link requires spatial lipidomics of paired human IPFP and synovium together with receptor-level functional experiments.

Extracellular vesicles may carry several classes of signals together. Vesicles from OA IPFP altered chondrocyte metabolism and senescence, with let-7b-5p and let-7c-5p implicated in experimental assays (23). These findings support an IPFP–cartilage route, while transfer across the IPFP–synovium interface remains unproven. The term “IPFP-derived vesicles” does not define a uniformly therapeutic or pathogenic product. Activity depends on donor disease status, cell source, isolation method, dosage, and recipient tissue. Protective stromal cell products (36, 37) can coexist with damaging OA tissue vesicles (23). Studies should report particle identity and cargo, compare conditioned medium after vesicle depletion, and confirm that recipient cells in the relevant anatomical location can internalize vesicles. **Table 1** summarizes the major components and evidence sources.

**Table 1:** Key components of the osteoarthritis IPFP–synovium interface.

Component	Major source	Effects at the interface	Study type and limitations
Macrophages	IPFP and synovium	Integrate lipid/damage cues; cytokines, chemokines, and stromal remodeling	Human single-cell/nucleus profiling (5, 6), flow cytometry and conditioned-medium assays (14). Cell states and responses are observed; necessity for sustained human interfacial signaling remains untested.
Fibroblasts/stromal cells	Synovium and IPFP	Immune recruitment, ECM remodeling, fibrosis, and adipogenic–fibroblast transitions	Human single-cell/nucleus and spatial profiling (5, 6); mouse single-cell analysis and lineage tracing (7). Shared programs support coordinated remodeling, while human lineage relationships remain inferred.
Adipocytes	IPFP	Adipokines, fatty acids, redox signals, and vesicle signals	Human single-nucleus/spatial profiling (5, 6), paired-tissue adipokine measurements (25), and high-fat-diet animal models (22). Bulk secretion and dietary interventions cannot isolate adipocyte-specific signals crossing the interface.
Leptin	Mainly adipocytes	May enhance inflammation and catabolic responses	Human tissue/fluid measurements (25), clinical associations (26, 27), and conditioned-medium blockade assays (28). Leptin alone did not explain secretome effects; local source and causal contribution remain uncertain.
Adiponectin	Adipocytes and other cells	Context- and isoform-dependent immune/stromal effects	Human tissue expression (25), cross-sectional fluid associations (26, 27), and conditioned-medium blockade assays (28). Severity associations do not establish causality, and adiponectin blockade did not explain the whole secretome.
IL-6/TNF/IL-1β	Multiple interface cell types	Stromal cell activation and inflammation amplification	Human tissue-derived macrophage and cytokine assays (14); conditioned-medium experiments in chondrocytes and synoviocytes (28). Mixed mediators prevent attribution to one cytokine or tissue source.
CCL2 and related chemokines	Stromal and immune cells	Monocyte recruitment and cell retention	Human synovial tissue/fluid and cultured-cell assays; genetic and pharmacological inhibition in mouse OA (16). Recruitment is supported in broader OA; interfacial necessity and the IPFP contribution remain untested.
Lipid mediators	Adipocytes, stromal cells, and immune cells	Inflammatory or pro-resolving macrophage/fibroblast remodeling	Human tissue/fluid fatty-acid profiling (32), lipidomics and production assays (33), and intervention in obesity-associated animal OA (35). Detection does not establish transfer; systemic context and handling affect abundance.
Extracellular vesicles	Multiple IPFP cell types	Transfer regulatory cargo, including microRNAs; alter recipient-cell metabolism	Human OA IPFP vesicles tested in chondrocytes, human/mouse cartilage explants and mouse OA (23); stromal-cell vesicles in macrophages and mouse OA (37). Endogenous interfacial transfer and human efficacy remain unproven.

Study types describe evidence sources, not a quality ranking. Whole-joint or cultured-cell effects do not establish communication across the intact human interface. ECM, extracellular matrix; IPFP, infrapatellar fat pad; OA, osteoarthritis.

## 5 From local crosstalk to whole-joint pathology

The IPFP–synovium loop can affect the whole joint through multiple pathways. Activated synovium releases cytokines and proteases into synovial fluid, altering chondrocyte metabolism and matrix turnover. Cartilage debris can stimulate synovial fibroblasts, contributing to inflammatory feedback within the joint (16, 38). Similar responses to damage-associated molecular patterns (DAMPs) may extend this loop to IPFP cells, but this route requires direct testing. Vascular and fibrotic remodeling may also alter tissue oxygenation and solute transport. The IPFP can amplify these processes even when they originate elsewhere.

Pain also links the IPFP and synovium. Both tissues are richly innervated, and inflammatory mediators can sensitize nociceptors or facilitate neuro-immune communication. Synovium from painful knee regions has distinct phenotypes and specific fibroblast subpopulations (17). Patients with knee OA and comorbid diabetes show more severe joint pain, synovitis, and synovial immune cell infiltration (39). Mechanical impact, fibrosis, and altered tissue stiffness can coexist with biochemical sensitization, so inflammatory secretions alone cannot account for pain. Effects on subchondral bone through synovial fluid and vascular pathways are mechanistically plausible, but direct evidence remains limited. The interface is best viewed as a local amplifier in whole-joint OA, with its importance likely varying among inflammatory, metabolic, post-traumatic, and mechanically driven phenotypes.

Imaging may eventually capture this biological process non-invasively, but current measurements reflect tissue structure and signal texture rather than cellular communication. MRI texture analysis of the IPFP can predict incident knee OA, and radiomic signatures have been developed for diagnosed disease (40, 41). Mechanical testing has also characterized the stiffness and time-dependent behavior of IPFP from patients with end-stage OA (42). These metrics might help select patients with an active interface phenotype for clinical trials if prospective studies link them to tissue or synovial fluid programs. Such links are needed before the measurements can be regarded as immunometabolic biomarkers.

## 6 Therapeutic implications

Therapeutic strategies should target pathological cellular states or cell-cell interactions rather than the IPFP as a whole (43). Macrophage-targeted approaches include blocking recruitment or specific cytokine programs, promoting inflammation resolution, and altering lipid processing. Intervention studies support targeting CCL2/CCR2, GM-CSF, and CCL17 in broader mouse OA models, but do not directly establish their roles in IPFP–synovium communication (16, 30, 31). Single-cell analyses of mouse post-traumatic OA synovium inferred macrophage colony-stimulating factor (M-CSF) signaling (44). That study did not test therapeutic blockade or cross-interface signaling. Broad macrophage depletion, however, could impair tissue-resident functions needed for homeostasis and repair. Fibroblast-directed strategies can target chemokine production, profibrotic transformation, or immune-stromal signaling. Metabolic interventions can modulate adipokine receptors, fatty acid flux, oxidative stress, or pro-resolving lipid pathways.

An association with inflammation does not, by itself, justify IPFP resection. The tissue has several biological functions: it helps distribute loads, provides vascular and neural support, and may facilitate stromal cell-mediated repair. Ovine experiments found changes in inflammatory and remodeling markers after IPFP resection, without detected morphological differences (45). A retrospective clinical study reported improvements in symptoms and MRI scores after partial resection, but its uncontrolled design does not establish disease-modifying effects (46). Products derived from IPFP-resident mesenchymal stromal cells have anti-inflammatory effects under certain preparation conditions (36, 37), whereas OA tissue-derived extracellular vesicles may be detrimental depending on donor status and vesicle cargo (23). This functional diversity favors selective modulation, local delivery, and biomarker-guided enrollment over nonspecific resection or inhibition.

These mechanisms are unlikely to dominate in every knee, so patient selection matters. Obesity-related OA with synovitis is a candidate, supported indirectly by reduced synovitis after resolvin D1 treatment in obese mice with surgically induced OA (35). Paired assessment of the IPFP and synovium could help test whether local changes in lipid handling accompany persistent inflammation. Metabolic syndrome-related or diabetic OA warrants consideration of systemic metabolic status alongside local tissue findings. Paired-tissue adipokine differences and increased synovial inflammation have been reported in these groups (25, 39). Experimental observations of coordinated IPFP–synovium remodeling and injury-associated synovial macrophage programs also support studying post-traumatic OA with persistent synovitis (7, 44). Recording the interval from injury would help distinguish persistent tissue changes from those observed during early recovery. These phenotypes overlap, and their dependence on the interface remains unvalidated. Obesity does not define a uniform IPFP phenotype (6). Future comparisons should assess local synovitis alongside the clinical phenotype and account for sex, medication use, and OA stage.

## 7 Discussion and future directions

Anatomical proximity, coordinated cellular remodeling, conditioned medium experiments, and intervention studies support the interface model, but the causal sequence remains uncertain. Most human samples come from joint replacement surgery and represent advanced, previously treated disease. Even high-resolution single-cell atlases provide cross-sectional snapshots. Ligand–receptor algorithms infer communication from expression, sometimes incorporating spatial proximity, but cannot demonstrate signal flux or necessity. Where ethically feasible, prospective studies should collect synovial fluid and image-guided tissue samples in early-stage OA and link these data to pain trajectories and quantitative structural outcomes.

Spatial tissue organization poses a further problem. Bulk tissue comparisons dilute local gradients, while tissue dissociation removes matrix-bound signals and tissue topology. Spatial transcriptomics, multiplexed protein imaging, and spatial lipidomics should map the synovial lining, IPFP fibrous septa, blood vessels, nerves, and transition zones within the same specimen. IPFP multi-omics datasets and emerging multi-tissue knee atlases from 2025 provide a basis for these studies (6, 47). Spatial predictions still need validation through perturbation experiments in human tissue explants or organotypic co-cultures. Sampling locations must be recorded precisely so that inflammatory gradients within the fat pad are not mistaken for inter-patient variability.

In capture-based spatial transcriptomics, RNA diffusion during permeabilization and spot swapping can blur tissue boundaries (48). In imaging-based platforms, segmentation errors can misassign transcripts to neighboring cells (49). Formalin fixation of rat liver and kidney caused lipid-class-dependent oxidation and hydrolysis, while paraffin processing depleted lipid signals and altered their spatial distributions (50). These findings need validation in joint tissues. Matched fresh-frozen samples, recorded fixation and handling times, and targeted mass-spectrometric confirmation can help distinguish biological gradients from processing artifacts.

Studies should place greater emphasis on cell states and cell-cell interactions than on cell abundance alone. Macrophage and fibroblast nomenclature should be standardized across datasets, with the underlying states validated through protein, metabolic, and functional assays. Stable isotope tracing can test whether IPFP-derived lipids reach synovial cells, while barcoded or microfluidic co-cultures can distinguish soluble, extracellular vesicle-dependent, and contact-dependent effects. Analyzing cartilage and synovial fluid together helps determine whether interfacial signaling remains local or propagates throughout the joint.

Clinical translation depends on validation at several levels. Exploratory datasets should identify spatially defined cell states that can be detected reproducibly in independent cohorts. Selective blockade in human tissue explants can then test the necessity and directionality of signals, and animal models should be chosen to reflect the relevant metabolic or post-injury context. Early-stage clinical trials should measure pharmacodynamic changes at the interface alongside pain scores. Negative results can also be informative if they show that a pathway is redundant, restricted to a specific disease stage, or active only within a particular molecular subset.

Four questions remain unresolved. It is unclear whether IPFP–synovium communication is necessary for sustained inflammation and, if so, at which disease stages. The IPFP-derived lipids or other mediators that reach synovial cells at active concentrations still need to be identified, as do the macrophage or fibroblast states that can be modified while preserving repair. A combined imaging and molecular signature also needs to be evaluated for its ability to predict treatment response beyond clinical variables. Addressing these uncertainties requires stage-specific experiments to establish the source and necessity of signals, followed by prospective validation. Current evidence favors viewing the adjacent IPFP and synovium as a dynamic immunometabolic interface in which pathological remodeling may amplify inflammation in specific knee OA phenotypes.
